# Remodeling of Oxidative Energy Metabolism by Galactose Improves Glucose Handling and Metabolic Switching in Human Skeletal Muscle Cells

**DOI:** 10.1371/journal.pone.0059972

**Published:** 2013-04-01

**Authors:** Eili Tranheim Kase, Nataša Nikolić, Siril Skaret Bakke, Kaja Kamilla Bogen, Vigdis Aas, G. Hege Thoresen, Arild Christian Rustan

**Affiliations:** 1 Department of Pharmaceutical Biosciences, School of Pharmacy, University of Oslo, Oslo, Norway; 2 Institute of Pharmacy and Biomedical Laboratory Science, Oslo and Akershus University College of Applied Sciences, Oslo, Norway; 3 Department of Pharmacology, Institute of Clinical Medicine, Faculty of Medicine, University of Oslo and Oslo University Hospital, Oslo, Norway; Pennington Biomed Research Center, United States of America

## Abstract

Cultured human myotubes have a low mitochondrial oxidative potential. This study aims to remodel energy metabolism in myotubes by replacing glucose with galactose during growth and differentiation to ultimately examine the consequences for fatty acid and glucose metabolism. Exposure to galactose showed an increased [^14^C]oleic acid oxidation, whereas cellular uptake of oleic acid uptake was unchanged. On the other hand, both cellular uptake and oxidation of [^14^C]glucose increased in myotubes exposed to galactose. In the presence of the mitochondrial uncoupler carbonylcyanide p-trifluormethoxy-phenylhydrazone (FCCP) the reserve capacity for glucose oxidation was increased in cells grown with galactose. Staining and live imaging of the cells showed that myotubes exposed to galactose had a significant increase in mitochondrial and neutral lipid content. Suppressibility of fatty acid oxidation by acute addition of glucose was increased compared to cells grown in presence of glucose. In summary, we show that cells grown in galactose were more oxidative, had increased oxidative capacity and higher mitochondrial content, and showed an increased glucose handling. Interestingly, cells exposed to galactose showed an increased suppressibility of fatty acid metabolism. Thus, galactose improved glucose metabolism and metabolic switching of myotubes, representing a cell model that may be valuable for metabolic studies related to insulin resistance and disorders involving mitochondrial impairments.

## Introduction

Cultured human skeletal muscle cells may constitute a valuable model system for studying muscle metabolism and metabolic disorders like insulin resistance and type 2 diabetes. However, when compared to biopsies, myotubes are less oxidative with reduced levels of cytochrome c oxidase and creatine kinase [1]. Several studies have shown that primary human myotubes preserve their *in vivo* metabolic phenotype *in vitro* [2]–[5]. They are highly glycolytic when grown in presence of glucose [1] and have a low mitochondrial oxidative capacity resembling fast (glycolytic) muscle fibers [6]. Ideally, an oxidative human skeletal muscle cell model that could resemble slow (oxidative) muscle fibers to a higher degree would be desirable.

The Warburg effect [7], [8] is a phenomenon, which was first observed in cancer cells, where cells generate energy through aerobic glycolysis instead of mitochondrial oxidative phosphorylation (OXPHOS), even though sufficient oxygen is present [9]. For studies involving oxidative energy metabolism in cell models, the Warburg effect is undesirable. It has been proposed that oxidation of galactose to pyruvate through glycolysis yields no net production of adenosine triphosphate (ATP) forcing cells to rely on mitochondrial OXPHOS to generate sufficient ATP for cell survival. Indeed, cancer cells grown in a medium where glucose was replaced with galactose showed an increased oxygen consumption rate [10], [11]. Aguer et al [12] have recently confirmed that myotubes differentiated in galactose also increased oxygen consumption rate and decreased anaerobic glycolysis, shown by reduced production of lactate. Myotubes grown with glucose may derive most of their energy from glycolysis despite aerobic culturing conditions. When the cells are grown in medium with galactose they will be forced to use oxidative phosphorylation to get sufficient ATP most likely from an increased glutaminolysis [13]. Therefore, in this study we aimed to remodel energy metabolism in myotubes by replacing glucose with galactose [10] in the culture medium, thereby circumventing the Warburg effect, to study how metabolism of glucose and fatty acids could be altered.

Human myotubes transport hexoses mainly by the basal glucose transporter GLUT1 and the insulin-sensitive transporter GLUT4. Both these transporters seem to have a higher affinity for glucose than for other hexoses [14]. To be metabolized, galactose has to be converted to glucose-6-phosphate [15]. Galactokinase and galactose-1-phosphate uridylyltransferase are the two first enzymes involved in this reaction pathway [15]. Both enzymes have been found to be active in skeletal muscle *in vivo* [16], but to our knowledge, they have not been detected in myotubes.

Skeletal muscle of healthy individuals is metabolically flexible and will easily switch from mainly lipid oxidation during fasting to glucose oxidation in the postprandial state [17]. Loss of this ability is termed metabolic inflexibility [17], and is linked to obesity and type 2 diabetes [18], [19]. A study by Ukropcova et al indicated that metabolic switching could be an intrinsic characteristic of human skeletal muscle cells [5]. They described metabolic switching *in vitro* in human myotubes as suppressibility, defined as the ability of acutely added glucose to suppress fatty acid oxidation, and adaptability [5], defined as the capacity of the cell to increase fatty acid oxidation upon increased fatty acid availability. Previously, we have shown that these *in vitro* metabolic parameters are influenced by pretreatment of the myotubes with e.g. n-3 fatty acids and hyperglycemia [20], [21].

The aim of this study was to exchange glucose for galactose in the growth and differentiation media of human myoblasts to explore if galactose, as a tool to enhance mitochondrial OXPHOS, could alter metabolism of glucose and fatty acids as well as modifying metabolic switching of the myotubes. We used labeled substrates such as oleic acid, glucose and galactose to study in detail the effects of galactose on glucose and fatty acid uptake and oxidation.

## Materials and Methods

### Materials

Dulbecco‘s modified Eagle‘s medium (DMEM-Glutamax™ without glucose, Ref 11966-025), foetal bovine serum, DMEM without phenol red, penicillin-streptomycin-amphotericin B, and trypsin-EDTA were obtained from Gibco, Life Technologies (Paisley, UK). Ultroser G was purchased from Pall Biosepra (Cergy-Saint-Christophe, France). [1-^14^C]oleic acid (55 mCi/mmol), D-[U-^14^C]glucose (2.9 mCi/mmol) and D-[1-^14^C]galactose (55.5 mCi/mmol) were purchased from PerkinElmer NEN® (Boston, MA, USA). Insulin Actrapid was from Novo Nordisk (Bagsvaerd, Denmark). Carbonylcyanide p-trifluormethoxy-phenylhydrazone (FCCP), oleic acid, bovine serum albumin (BSA) (essentially fatty acid-free), extracellular matrix (ECM) gel were purchased from Sigma-Aldrich (St. Louis, MO, US). Glass bottom plates were purchased from MatTek (Ashland, MA, US). RNeasy Mini kit and RNase-free DNase were purchased from Qiagen Sciences (Oslo, Norway). Agilent Total RNA isolation kit was from Agilent Technologies (Santa Clara, CA, US). The primers were purchased from Invitrogen (Paisley, Scotland, UK), while SYBR® Green and TaqMan® reverse-transcription reagents kit and TaqMan® Low Density Custom Arrays were from Applied Biosystems (Foster City, Canada). Hydrophobic MultiScreen® HTS plates were from Millipore (Billerica, MA, US). Corning® CellBIND® tissue culture plates were obtained from Corning Life-Sciences (Schiphol-Rijk, The Netherlands). OptiPhase Supermix and UniFilter®-96 GF/B were delivered by PerkinElmer (Shelton, CT, US). MitoTracker®Red FM, Bodipy 493/503 (4,4-difluoro-1,3,5,7,8-pentamethyl-4-bora-3a,4a-diaza-s-indacene) and Hoechst 33258 were obtained from Molecular Probes, Invitrogen (Carlsbad, CA, US). Nitrocellulose membrane was from Amersham™ Hybond™-ECL (GE Healthcare, US), antibodies recognizing myosin, slow muscle (MAB1628) was from Millipore, pyruvate dehydrogenase 4 (PDK4, H00005166-A02) from Abnova, Mitoprofile® Total OXPHOS WB antibody cocktail (ab110411) from Abcam, human total (mAB#2532) and phosphorylated (mAB#2531) AMP-activated protein kinase (AMPK) and β-actin (mAB#4970) from Cell Signaling Technology Inc. (Beverly, MA, US). Immun-Star TM Western C TM Kit, Laemmli buffer, Tris/glycine buffer, Mini-Protean® TGX™ gels and protein assay reagent were purchased from BioRad (Copenhagen, Denmark). All other chemicals used were standard commercial high purity quality.

### Ethics Statement

The biopsies were obtained with informed written consent and approval by the Regional Committee for Medical and Health Research Ethics (Oslo, Norway). The research performed in this study was approved, as a part of a larger project, by the Regional Committee for Medical and Health Research Ethics (Oslo, Norway).

### Culturing of Human Myotubes

Satellite cells were isolated as previously described [22] from the *M. obliquus internus abdominis* from healthy volunteers. Donors were 49±14 (mean, SD) years old, had a body mass index of 24.0±4.0 kg/m^2^, fasting glucose 5.2±0.7 mM, plasma lipids and blood pressure within normal range, and no family history of diabetes. The cells were cultured in DMEM-Glutamax-I supplemented with 5.5 mM glucose or 5.5 mM galactose, 2% foetal bovine serum, 2% Ultroser G, penicillin (100 units/ml) and streptomycin (100 µg/ml), amphotericin B (1.25 µg/ml) and 5.5 mM sodium pyruvate for proliferation. At 70–80% confluence the growth medium was replaced by DMEM-Glutamax-I supplemented with 5.5 mM glucose or 5.5 mM galactose, 2% foetal bovine serum, penicillin (100 units/ml), streptomycin (100 µg/ml), amphotericin B (1.25 µg/ml), 5.5 mM sodium pyruvate and insulin (25 pM) to induce differentiation. The cells were cultured in humidified 5% CO_2_ atmosphere at 37°C, and the medium was changed every 2–3 days. Experiments were performed after 7 days of differentiation.

### RNA Isolation and Analysis of Gene Expression by qPCR

After proliferation and differentiation in DMDM-media with 5.5 mM glucose or 5.5 mM galactose, cells were harvested and total RNA was isolated by RNeasy Mini kit (Qiagen Sciences, Oslo, Norway) according to the supplier’s total RNA isolation protocol. Equal amount of RNA obtained from myotubes from different donors were reversely transcribed with a High Capacity cDNA Archive Kit. Total RNA (1 µg/µl) was reversely transcribed with hexamere primers using a PerkinElmer Thermal Cycler 9600 (25°C for 10 min, 37°C for 1 h, 99°C for 5 min) and a TaqMan reverse-transcription reagents kit (Applied Biosystems). Primers (36B4, CPT1b, CYC1, GALK1, GALK2, GALT, GAPDH, HKII, MYH2, MCAD, PDK4, SLC2A1, SLC2A4) were designed using Primer Express® (Applied Biosystems). Primer sequences are available upon request. Each target gene were quantified in triplicates and carried out in a 25 µl reaction volume according to the supplier’s protocol. All assays were run for 40 cycles (95°C for 12 s followed by 60°C for 60 s). The transcription levels were normalized to the reference control gene 36B4.

### Western Blot Analysis

After proliferation and differentiation in DMEM-media with 5.5 mM glucose or 5.5 mM galactose, cells were harvested in Laemmli buffer. Total cell lysates were electrophoretically separated on 4–20% Mini-Protean® TGX™ gels with Tris/glycine buffer (pH 8.3) followed by blotting to nitrocellulose membrane and incubation with antibodies recognizing human total and phosphorylated AMP-activated protein kinase (AMPK), myosin, slow muscle, pyruvate dehydrogenase 4 (PDK4), β-actin and an OXPHOS antibody cocktail recognizing complex I subunit NDUFB8, complex II subunit, complex III subunit core 2, complex IV subunit II and ATP synthase subunit. Immunoreactive bands were visualized with enhanced chemiluminescence and quantified with Image lab (version 4.0) software. Antibody against β-actin was used to normalize the protein-antibody signal versus the amount of protein loading.

### Staining and Live Imaging of Neutral Lipids and Mitochondria in the Cells

Myotubes were cultured as described above, with the exception of using 12-well glass bottom plates, coated with extracellular matrix gel. The cells were proliferated and differentiated in DMEM-media with 5.5 mM glucose or 5.5 mM galactose. Myotubes were incubated in 37°C and 5% CO_2_ with Bodipy 493/503 (2.0 µg/ml) for 5 min to stain neutral lipids, Hoechst 33258 (2.5 µg/ml) for 15 min to stain nuclei and MitoTracker®Red FM (100 nM) for 15 min to stain mitochondria.

Automated image acquisition was performed in culture medium without phenol red with a Scan^∧^R platform (Olympus IX81 inverted fluorescence microscope) equipped with a temperature and CO_2_-enrichment incubator for long-term live imaging, as described by Hessvik et al [21]. We used a 20× objective and live images of myotubes were taken at 25 positions per well. The background-subtracted maximal intensity projection from 7 images taken in z-direction (1 µm apart) was used for each color channel at each position. Image acquisition was carried out for about 1 h.

Scan^∧^R software was used for automated image analysis, using edge detection algorithm for object segmentation to quantify the mitochondrial content (intensity of MitoTracker®Red FM), and neutral lipid content (intensity of Bodipy 493/503), and number of nuclei per image. After gating out aggregates and dead cells, in average 71 images per parameter were analyzed with an average of 29±4 nuclei per image.

### Radiolabeled Tracer Studies

Uptake and oxidation of oleic acid (OA), glucose and galactose were measured as previously described [23]. Briefly, after myotubes had grown in DMEM-media with 5.5 mM glucose or 5.5 mM galactose, cells underwent CO_2_ trapping for 4 h with [^14^C]oleic acid (1 µCi/ml, 100 µM) or [U-^14^C]glucose (1 µCi/ml, 100 µM) with or without the mitochondrial uncoupler carbonylcyanide p-trifluormethoxy-phenylhydrazone (FCCP) or [1-^14^C]galactose (1 µCi/ml, 100 µM). Myotubes were harvested in 0.1 M NaOH. CO_2_ and cell-associated radioactivity was measured by liquid scintillation, and protein content was determined according to Bradford [24]. Uptake and oxidation of radiolabeled substrate were related to cell protein content and given as nmol/mg protein. Suppressibility, defined as the ability of the cells to decrease oleic acid oxidation by acutely added glucose (5 mM), was calculated as: [(1-(oxidation of oleic acid at 5 mM glucose/oxidation of oleic acid at no glucose added))×100%], and adaptability, defined as the ability of the cells to increase oleic acid oxidation with increasing oleic acid concentration, was calculated as: [oxidation of 100 µM oleic acid/oxidation of 5 µM oleic acid] as previously described [21].

For lipogenesis, cells were incubated in DMEM-media (without glucose or galactose) with [U-^14^C]glucose (1 µCi/ml, 200 µM) or [1-^14^C]galactose (1 µCi/ml, 200 µM) for 24 h and harvested in dH_2_O, assayed for protein [24], and total lipids were isolated by filtration of the cell lysate through hydrophobic MultiScreen® HTS plates (Millipore, Billerica, MA, USA). The amount of lipids was determined by liquid scintillation counting, and lipogenesis was related to cell protein content and given as nmol/mg protein.

### Presentation of Data and Statistical Analysis

Data in text and figures are given as mean (±SEM) from n = number of separate experiments, all performed on muscle cells established from separate cell donors. At least 3 parallels were included in each experiment. For the data from the live cell imaging experiments, one separate culture well was included per donor (n = 3). Comparisons of different treatments were evaluated by two-tailed, paired Student’s t-test, and *P*<0.05 was considered significant.

## Results

### Galactose is Utilized by Myotubes to a Lesser Extent than Glucose

**Figure 1:**
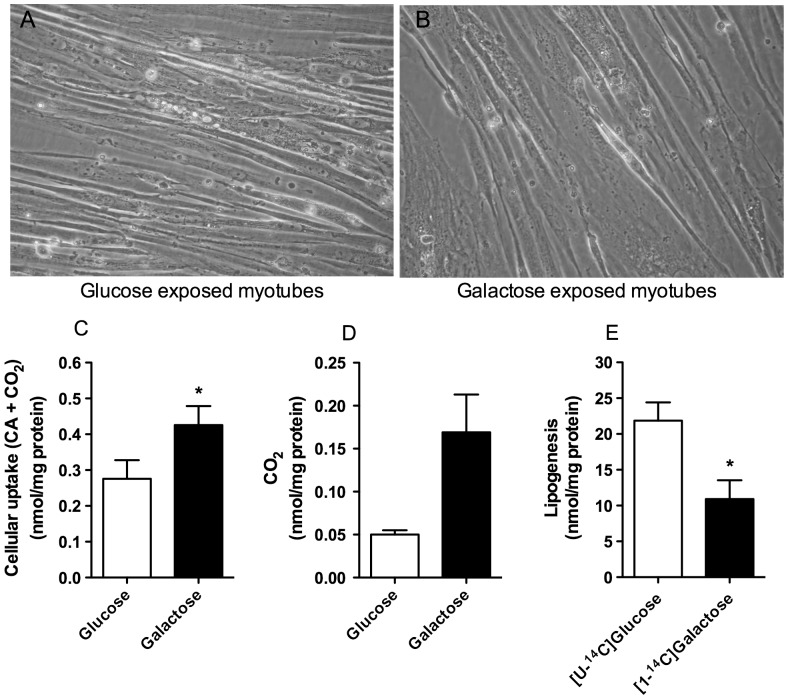
Cellular handling of labeled galactose. Myotubes were grown in DMEM-media with 5.5 mM glucose (**A**) or 5.5 mM galactose (**B**). For cellular uptake (n = 4) (**C**) and oxidation (CO_2_-trapping) (n = 4) (**D**) the cells were exposed to [1-^14^C]galactose (1 µCi/ml, 200 µM) for 4 h before harvesting. For lipogenesis (n = 6) (**E**) the cells were exposed to [U-^14^C]glucose (1 µCi/ml, 200 µM) or [1-^14^C]galactose (1 µCi/ml, 200 µM) for 24 h before harvesting as described in Materials and Methods. Values represent nmol/mg cell protein given as means ± SEM. *P<0.05 vs. glucose pretreatment.

Using light microscopy we observed that myotubes that received galactose appeared morphologically thinner and grew and differentiated at a slower rate than cells grown in presence of glucose, spending 2 or 3 days extra to reach 70–80% confluence (figure 1A and B). The mean total cell protein content per well at time of harvest was the same regardless which carbohydrate used during growth and differentiation (data not shown).

Galactose is not a preferred endogenous energy source for myotubes, so we wanted to explore whether galactose could be taken up and metabolized by the cells. When the cells were grown in glucose during proliferation and differentiation, 0.28 nmol/mg protein ^14^C-galactose was taken up by the cells (figure 1C) and 0.05 nmol/mg protein or about 20% was oxidized (figure 1D). This was about 1–2% of what was observed taken up and oxidized for ^14^C-glucose. For cells grown in presence of galactose, 0.43 nmol/mg protein ^14^C-galactose was taken up (figure 1C) and 0.17 nmol/mg protein or about 40% was oxidized (figure 1D). Although myotubes generally show a low rate of lipogenesis [25], [26], labeled galactose was incorporated into complex lipids in cells grown in presence of galactose but significantly less than observed when using labeled glucose (figure 1E).

### Galactose Induced an Increase in Oxidation of Oleic Acid

**Figure 2:**
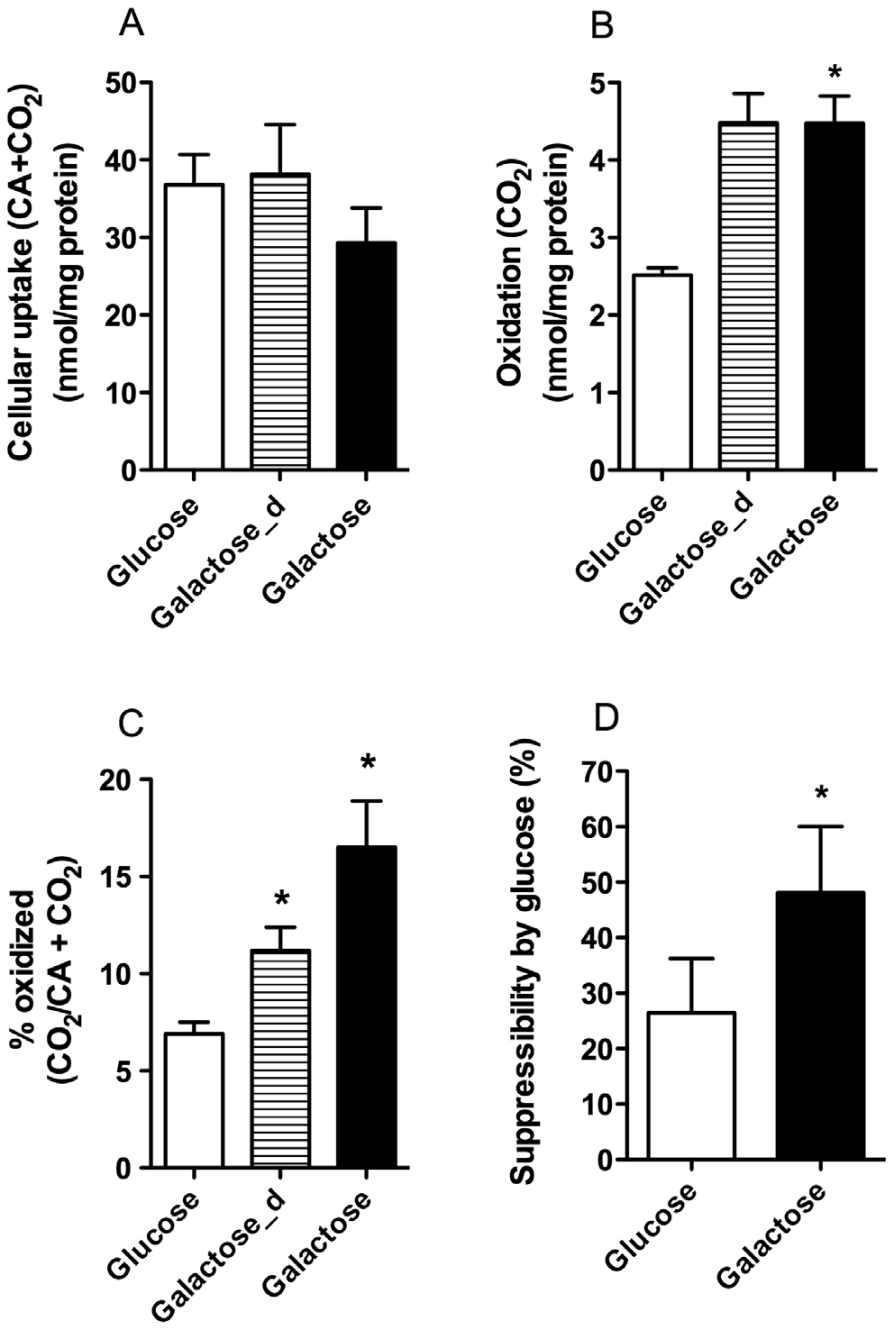
Effect of galactose treatment on oleic acid metabolism. Myotubes were either grown in DMEM-media with 5.5 mM glucose or 5.5 mM galactose during the whole seeding period, or with 5.5 mM glucose during proliferation and 5.5 mM galactose during differentiation (Galactose_d). Thereafter, the cells were exposed to [1-^14^C]oleic acid (1 µCi/ml, 100 µM) for 4 h as described in Materials and Methods. The figures show cellular uptake (n = 6) (**A**), oxidation (n = 6) (**B**), % oxidized (CO_2_/CA+CO_2_) (n = 6) (**C**) and suppressibility (n = 3) (**D**) of [1-^14^C]oleic acid. Suppressibility, defined as the ability of the cells to decrease oleic acid oxidation by acutely added glucose, was calculated as: [(1-(oxidation of oleic acid at 5 mM glucose/oxidation of oleic acid at no glucose added))×100%]. Values represent means ± SEM. *P<0.05 vs. glucose.

We next wanted to explore whether galactose exposure during both proliferation and differentiation of the cells, or only during differentiation, could improve the oxidative potential and cellular handling of fatty acids. Galactose pretreatment of the cells, regardless of time with galactose, did not significantly alter cellular uptake of ^14^C-oleic acid (OA) (figure 2A). Only galactose pretreatment during both proliferation and differentiation significantly increased oxidation of OA (1.8-fold, figure 2B) compared to glucose media. About 17% of labeled cellular OA was oxidized after exposure to galactose during both proliferation and differentiation compared to 7% after exposure to glucose (figure 2C). Oxidative reserve capacity (calculated as oxidation in presence of the mitochondrial uncoupler carbonylcyanide p-trifluormethoxy-phenylhydrazone (FCCP) minus basal oxidation) for OA was unchanged after treatment with galactose (data not shown). Compared to glucose exposure, the relative oxidation (figure 2C) was more increased when myotubes had been exposed to galactose exclusively during both proliferation and differentiation. Therefore, in the following experiments the cells received either glucose or galactose during the whole culturing period.

To investigate whether galactose could affect metabolic switching of myotubes, we measured suppressibility defined as the ability of myotubes to decrease oleic acid oxidation by acute addition of glucose (5 mM) [5]. The results showed a 2-fold increase in suppressibility with galactose as main carbohydrate source versus glucose (figure 2D). There was no effect of galactose supplementation on fatty acid adaptability (data not shown).

### Galactose Induced an Increase in Oxidative Metabolism of Glucose

**Figure 3:**
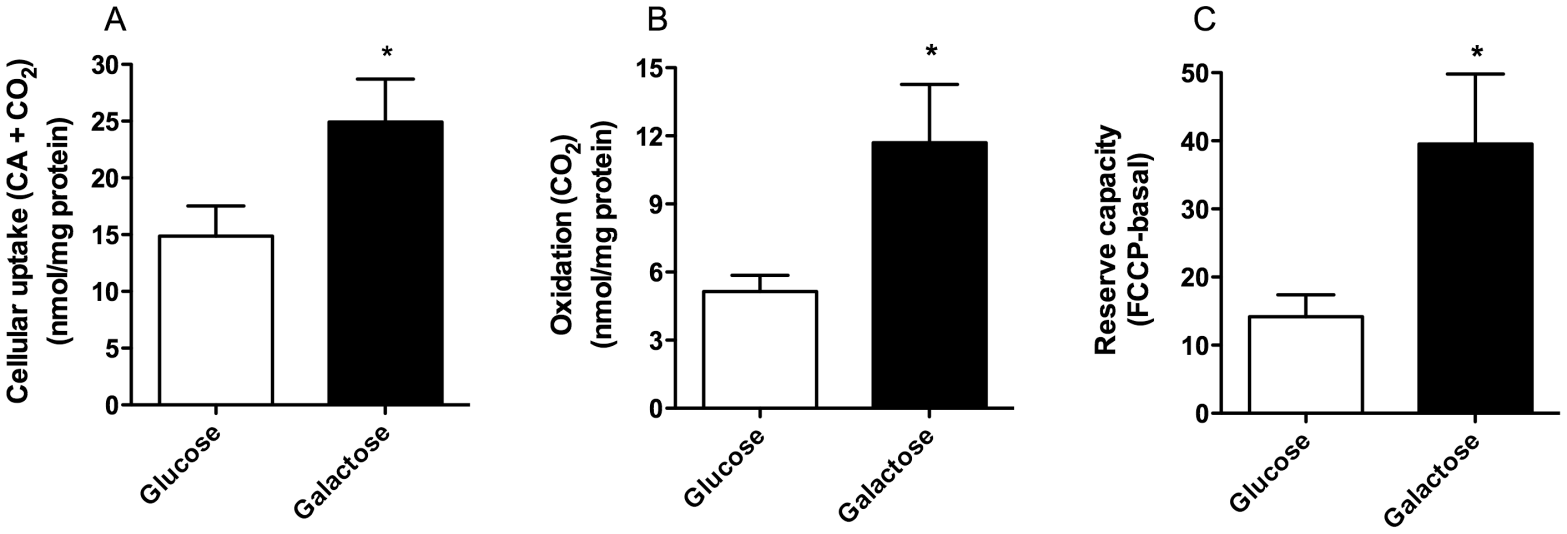
Effect of galactose treatment on glucose metabolism. Myotubes were grown in DMEM-media with 5.5 mM glucose or 5.5 mM galactose. Thereafter, the cells were exposed to [U-^14^C]glucose (1 µCi/ml, 100 µM) for 4 h as described in Materials and Methods. The figures show cellular uptake (n = 5–6) (**A**), oxidation (n = 5–6) (**B**) and reserve capacity (oxidation with FCCP (1 µM) – basal oxidation) (n = 3) (**C**) of [U-^14^C]glucose. Values represent means ± SEM. *P<0.05 vs. glucose.

We also wanted to explore whether galactose exposure could alter myotubes handling of labeled glucose. Galactose pretreatment increased both cellular uptake (figure 3A) and oxidation (figure 3B) of ^14^C-glucose 1.8-fold and 2.6-fold, respectively, compared to regular glucose media. Oxidative reserve capacity (FCCP - basal) for glucose (figure 3C) was also increased 3-fold after pretreatment with galactose. Thus, galactose-treatment of myotubes increased oxidative potential for glucose as well as promoted an increased glucose uptake by the cells.

To evaluate the impact of galactose on substrate preference for oxidation, we calculated that glucose oxidation relative to oleic acid oxidation was increased by 2.1-fold and 2.6-fold under basal conditions after glucose vs. galactose exposure, respectively. In the presence of FCCP (total CO_2_ formation) glucose oxidation relative to oleic acid oxidation for glucose vs. galactose exposure, was increased 2.7-fold and 6.0-fold, respectively (calculated from data presented in figures 2 and 3).

### Gene Expression of Pyruvate Dehydrogenase Kinase 4 was Reduced by Galactose

**Figure 4:**
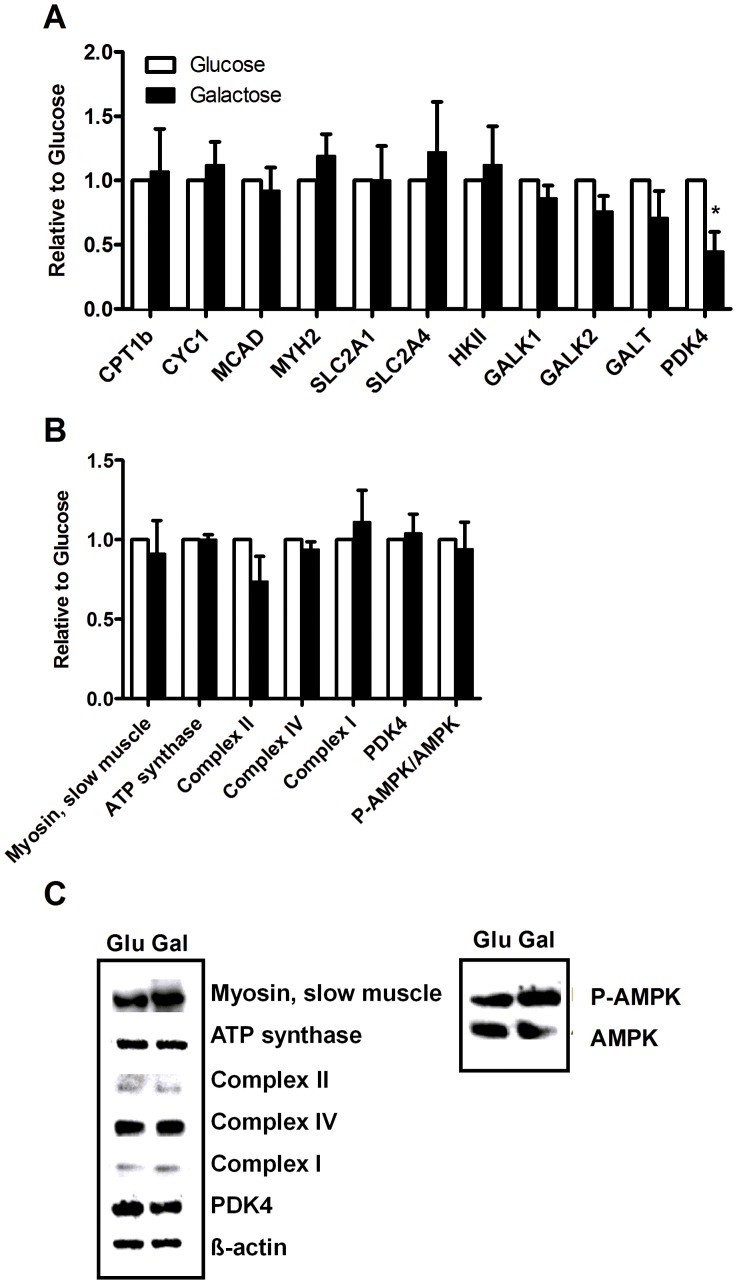
Effect of chronic galactose treatment on gene and protein expressions. Myotubes were grown in DMEM-media with 5.5 mM glucose or 5.5 mM galactose. Total RNA was isolated from the cells and analyzed by qPCR, while protein samples were harvested analyzed as described in Materials and Methods. Gene expressions were normalized to 36B4 and protein expressions to β-actin, except phosphorylated AMP-activated protein kinase (p-AMPK), which were normalized to total AMPK. Values in A and B represent fold change of genes/proteins in galactose-treated myotubes relative to glucose-treated myotubes, given as means ± SEM (n = 5). (**A**) Genes analyzed; CPT1b, carnitine palmitoyltransferase-1b; CYC1, cytochrome C; MCAD, acyl-coenzyme A-dehydrogenase; MYH2, myosin heavy chain 2, SLC2A1 and SLC2A4; glucose transporter 1 and 4, HKII; hexokinase II, GALK1 and 2; galactokinase 1 and 2, GALT; galactose-1-phosphate uridylyltransferase, PDK4; pyruvate dehydrogenase kinase 4. (**B**) Protein expression of myosin, ATP synthase subunit, slow muscle, complex II subunit, complex IV subunit II, complex I subunit NDUFB8, PDK4; pyruvate dehydrogenase 4, P-AMPK and AMPK. (**C**) Representative corresponding Western blots.

To explore gene regulatory effects of galactose mRNA analysis was performed by qPCR (figure 4A). Expression of genes important for fatty acid oxidation (carnitine palmitoyltransferase-1b; CPT1b), mitochondrial content (cytochrome C; CYC1, medium-chain acyl-coenzym A dehydrogenase; MCAD), fast muscle fiber type (myosin heavy chain 2; MYH2), glucose metabolism (glucose transporter 1 and 4; SLC2A1 and SLC2A4, pyruvate dehydrogenase kinase 4; PDK4) and galactose metabolism (hexokinase II; HKII, galactokinase 1 and 2; GALK1 and 2, galactose-1-phosphate uridylyltransferase; GALT) were studied. GALK1 and 2, and GALT were all expressed in myotubes, but not regulated by galactose. The only gene that responded to galactose exposure was PDK4 that was significantly decreased by about 50%. We further performed western analysis on total and phosphorylated AMP-activated protein kinase (AMPK), myosin, slow muscle, PDK4 and OXPHOS proteins with an antibody cocktail recognizing complex I subunit NDUFB8, complex II subunit, complex III subunit core 2, complex IV subunit II and ATP synthase subunit (figure 4B–C). However, we were not able to detect complex III subunit core 2, neither could we observe any significant differences between the detected protein expressions after galactose and glucose exposure.

### Galactose Induced an Increase in Mitochondrial and Neutral Lipid Contents

**Figure 5:**
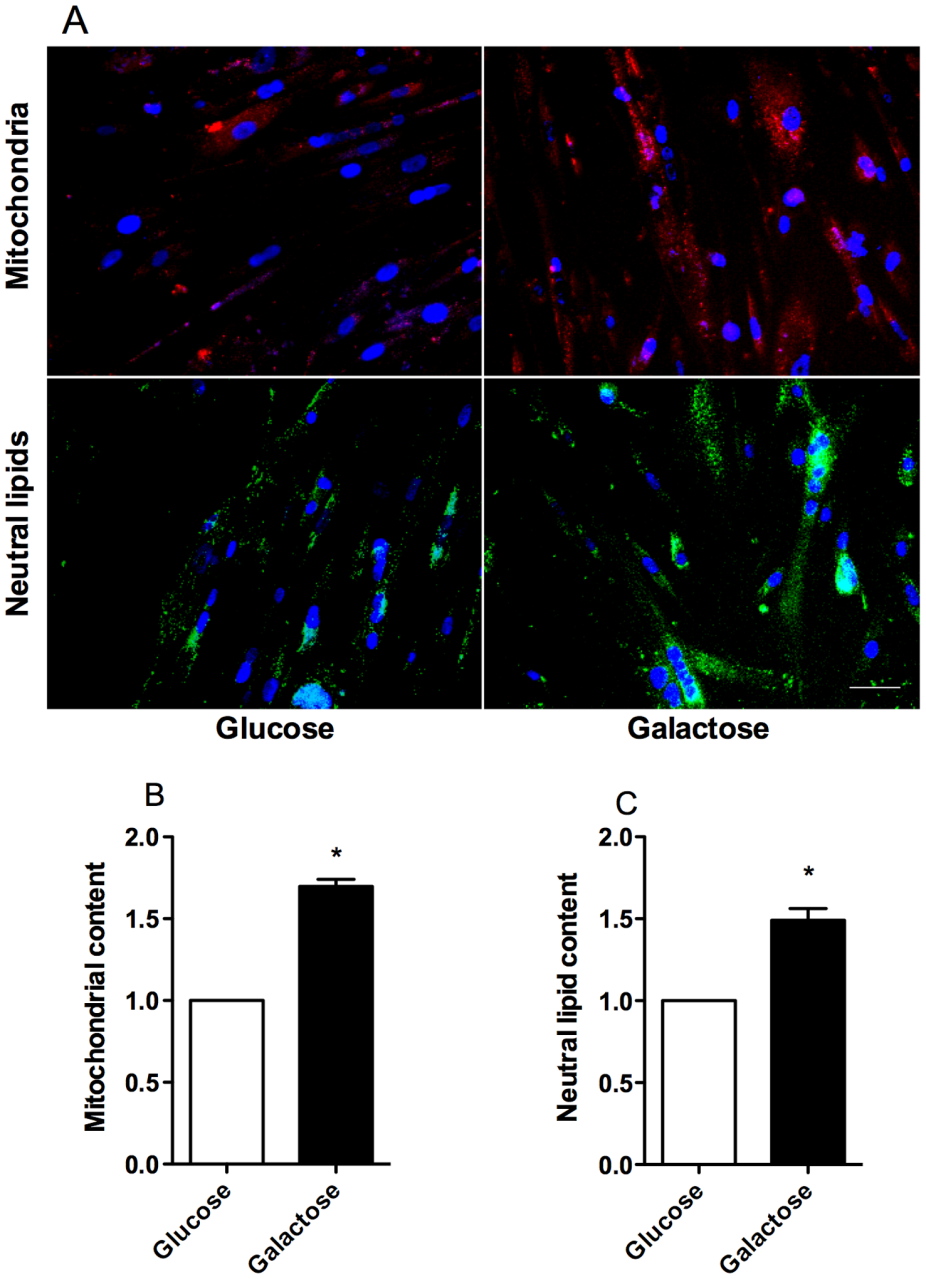
Effect of galactose treatment on mitochondrial and neutral lipid content. Myotubes were grown in DMEM-media with 5.5 mM glucose or 5.5 mM galactose. The cells were stained for mitochondria, neutral lipid and nuclei as described in Materials and Methods. The figures show (**A**) pictures of stained myotubes with mitochondria (red), neutral lipids (green) and nuclei (blue), (**B**) mitochondrial content, (**C**) neutral lipid content. Results represent fold change relative to glucose given as means ± SEM, (n = 3) and the data are normalized to number of nuclei. *P<0.05 vs. glucose. Arbitrary units for mitochondrial content per nucleus and neutral lipid content were 552232±137107, 860881±218244, respectively for glucose and 942449±71302, 1299938±102390, respectively for galactose.

Staining and quantification by live cell imaging was performed to explore whether galactose could alter the contents of mitochondria and neutral lipid in myotubes (figure 5A). Both the mitochondrial content (figure 5B) and the amount of neutral lipids (figure 5C) were increased by 65% and 45%, respectively, after exposure to galactose compared to glucose.

## Discussion

This study aimed to remodel energy metabolism in myotubes by replacing glucose with galactose during growth and differentiation to ultimately examine the consequences for fatty acid and glucose metabolism. We observed that oxidations of ^14^C-glucose and ^14^C-oleic acid were markedly increased. Acute treatment with a mitochondrial uncoupler (FCCP) showed an increased reserve capacity for glucose oxidation in galactose-exposed cells. Staining and live imaging of the cells showed that galactose promoted an increase in mitochondrial content compared to glucose. In parallel with increased glucose oxidation after exposure to galactose, we also observed an increased glucose uptake whereas the gene expression of PDK4 was reduced. Moreover, we found an increased suppressibility of oleic acid oxidation compared to cells grown in regular glucose-containing media.

Galactose is known from different cell systems to enhance mitochondrial respiration possibly by enhancing mitochondrial oxidative phosphorylation [10]–[12]. We observed that labelled galactose was taken up and oxidized to a much lower extent than glucose, despite the fact that they are both hexoses and are taken up in myotubes by the same transporters (GLUT1 and GLUT4). This difference can be due to that both transporters might have higher affinity for glucose than for other hexoses and that the kinetics may vary [14]. Further, cells grown with glucose may derive most of their energy from glycolysis despite aerobic culturing conditions, as can be shown by increased formation of lactate [12]. Hence, the reduced uptake and metabolism of galactose will presumably force the cells towards an increased mitochondrial ATP production using L-glutamine as energy substrate (glutaminolysis) for survival.

Aguer et al [12] recently observed that exposure to galactose during differentiation enhanced basal aerobic metabolism through measurements of oxygen consumption rate (OCR) in cells from post-diabetic subjects and their controls. They also showed that anaerobic glycolysis was decreased, demonstrated by reduced production of lactate probably caused by an increased production of pyruvate through galactose metabolism. We examined glucose replacement both during proliferation and differentiation and only during differentiation [12], and observed that oleic acid oxidation was even more enhanced relatively to cellular fatty acid uptake when glucose was replaced during the whole culturing period (figure 2C). The present study confirmed that galactose induced an increase in oxidative metabolism in the myotubes both in absence and presence of FCCP (figure 3). Furthermore, the observed increase in oxidative metabolism by the cells was mainly reflected as an increased glucose oxidation. The increased reserve capacity for glucose oxidation after galactose treatment may imply an increased mitochondrial capacity. This result was also supported by an increased mitochondrial content in galactose-treated cells detected by live cell imaging (figure 4B). We showed that galactose increased the total oxidative capacity of the myotubes for both OA and glucose, but mostly for glucose. Thus, galactose increased the total oxidative capacity of the cells, with a switch towards a preference for oxidative metabolism of glucose. In parallel with increased glucose oxidation we also observed an increased glucose uptake (figure 3A), which suggest that oxidative capacity is the driving force for increased glucose uptake. These results were supported by decreased mRNA level of PDK4 after galactose exposure. PDK4 inhibits by phosphorylation the activity of pyruvate dehydrogenase complex (PDC), which is crucial for maintaining the cells energy balance by regulating ATP levels [27]. PDK4 is negatively regulated by increased levels of pyruvate, NAD^+^ and ADP [27]. Based on these results, it seems that galactose forces the cells towards a state of energy deprivation where levels of ADP and pyruvate increases, expression of PDK4 is reduced, activity of PDC and the TCA cycle are increased so that labelled glucose or fatty acids are more efficiently metabolized by the galactose “activated” myotubes. Unfortunately, the protein expression of PDK4 was unchanged and did not support the mRNA results.

It has previously been observed that treatment of C2C12 muscle cells with weak uncouplers of oxidative phosphorylation (OXPHOS) enhanced glucose uptake [28]. Moreover, improvement of glucose metabolism together with increased oxidative capacity of myotubes have been demonstrated by electrical pulse stimulation of myotubes and by activation of cAMP/PKA and Ca^2+^ signaling pathways [29], [30]. Further, the observed increase in uptake and oxidation of glucose after exposure to galactose *in vitro* were positively correlated to fasting plasma glucose *in vivo*, but not when the cells were exposed to glucose (figure 3D). This suggests that certain traits in glucose metabolism from the *in vivo* phenotype can be detected in myotubes *in vitro* after remodelling by galactose. However, studying the mechanism for this observation was beyond the scope of the present study and needs to be further investigated.

Metabolic inflexibility is defined as loss of the ability to switch from mainly fatty acid oxidation during fasting to glucose oxidation in the postprandial state [17] and is linked to obesity and type 2 diabetes [18]. A study by Ukropcova et al [5] indicated that metabolic switching could be an intrinsic characteristic of human skeletal muscle cells. We recently showed that metabolic switching of human myotubes could be changed by alterations of the extracellular milieu [20], [21], [25], [31]. For instance, we have shown that the n-3 fatty acid eicosapentaenoic acid increased suppressibility of oleic acid oxidation by glucose [21] and at the same time increased glucose uptake and oxidation similar to observations with galactose in this study [32]. It should be noted that metabolic switching of the cells could also be a consequence of the expected response to energy deprivation and not mechanistically related to the suppressibility described in the studies above. Thus, regardless of mechanism, we here demonstrate that by supplementing the cells with galactose as energy source instead of glucose during growth and differentiation, we can improve metabolic switching of the myotubes by creating a more mitochondrial active cell model promoting increased glucose uptake and metabolism (figures 2D and 3).

Galactose treatment also increased the amount of neutral lipids in the cells (figure 5C). Increased storage of lipids together with improved glucose metabolism and metabolic switching of the cells has also been observed in other studies with human myotubes [21], [30], [32]. The ability to increase formation of neutral lipids and to increase oxidation of fatty acids after exposure to galactose may channel lipids away from generation of ‘‘lipotoxic’’ intermediates [33], [34].

A recent study [12] demonstrated that galactose treatment of myotubes had no effect on protein levels of mitochondrial markers (complex III, complex IV, succinate dehydrogenase) assessed by western blotting and that galactose did not have any negative effects on cellular physiology and metabolism [12]. Similarly, the results obtained on energy metabolism in the present study were not mirrored by changes in gene or protein expression. Some relevant genes (figure 4A) and proteins (figure 4B and C) important for mitochondrial oxidation, fatty acid, glucose and galactose metabolism were not regulated by galactose pretreatment, exept for mRNA for PDK4, supporting that the effects in this study on oxidative metabolism might be caused by post-translational mechanisms.

Taken together, this study showed that by removing glucose and introducing galactose as carbohydrate source during growth and differentiation, myotubes became generally more oxidative, seemed to utilize glucose better than oleic acid, which suggest an improved metabolic switching. Thus, galactose promoted a more oxidative skeletal muscle cell model that may be valuable for metabolic studies related to insulin resistance and disorders involving mitochondrial impairments.
